# Simulated patient methodology applied in health services research: a scoping review

**DOI:** 10.1186/s12913-026-14407-3

**Published:** 2026-03-26

**Authors:** Alexander Pachanov, Nadja Könsgen, Jessica Breuing, Barbara Prediger, Anna-Maria Zeitler, Dawid Pieper

**Affiliations:** 1 Faculty of Health Sciences Brandenburg, Brandenburg Medical School (Theodor Fontane), Institute for Health Services and Health System Research, Seebad 82/83, 15562 Rüdersdorf bei Berlin, Germany; 2 Center for Health Services Research, Brandenburg Medical School (Theodor Fontane), Rüdersdorf, Germany; 3 Institute for Research in Operative Medicine, Witten/Herdecke University, Cologne, Germany

**Keywords:** Simulated patients, Mystery clients, Pseudo customer study, Simulated patient methodology, Health services assessment

## Abstract

**Background:**

The simulated patient (SP) methodology is a valuable tool in health services research, allowing for the assessment of healthcare providers’ (HCPs) real-world behavior and performance through covert interactions. Although several evidence syntheses have examined its application in pharmaceutical settings, there is a gap in synthesizing recent research findings in other healthcare disciplines. Therefore, we aimed to map the application of the SP method in non-pharmaceutical healthcare settings.

**Methods:**

We conducted a scoping review, following the updated JBI (formerly Joanna Briggs Institute) guidance for scoping reviews. In October 2023, we searched MEDLINE (PubMed) and Embase (Elsevier) databases. We focused on peer-reviewed journal reports of primary studies of any design, published from May 2004 onwards, in English or German, that covertly utilized SPs in non-pharmaceutical healthcare settings. Two reviewers independently screened records and reports. Data extraction was conducted by one reviewer and validated by another one. We presented findings in table and diagram formats, accompanied by a narrative summary.

**Results:**

Our search identified 1,796 records, with 163 reports meeting the eligibility criteria. Between 2004 and 2023, the use of SP methodology in non-pharmaceutical healthcare settings increased, with nearly twice as many reports published between 2014 and 2023 compared to the previous decade. The median number of SP-HCP contacts was 202.5, with a median of eight SPs per study. Most reports originated from North America (63.8%), followed by Africa and Asia (each 12.9%). Interactions primarily occurred in clinics (39.9%) and practices (36.8%), with face-to-face (58.9%) and telephone (42.9%) being the most common modes of interaction. 48.5% of reports indicated that HCPs were informed of covert assessments by SPs before the study began. Many reports lacked details on HCP specialties (41.1%) and professions (46.0%). Post-study feedback to HCPs was rarely reported (7.4%), and ethics approval processes varied, with 12.3% of reports not addressing them at all.

**Conclusions:**

Over the past two decades, the SP methodology has been widely adopted across various regions and settings. However, inconsistent reporting and varying ethical considerations highlight the need for clearer guidelines to enhance the reliability and educational value of SP studies, ultimately leading to improved HCP practices and patient care quality.

**Scoping review registration:**

https://osf.io/8z9tn .

**Supplementary Information:**

The online version contains supplementary material available at 10.1186/s12913-026-14407-3.

## Background

The simulated patient (SP) methodology emerged in the 1960s as an educational tool in healthcare. SPs are trained individuals who enact a pre-defined scenario to assess the knowledge and skills of healthcare professionals (HCPs) during training or exams. This approach provides a safe environment for healthcare students to develop and enhance their clinical and communication skills, effectively preparing them for actual clinical situations [1]. Beyond education, SPs also play an important role in health services research. Guided by researchers, SPs covertly interact with HCPs (e.g., through visits, phone calls) to evaluate their behavior and performance in real-world settings.

Since 1973, when the first SP study was conducted [2], the SP method has been widely used in health services research, particularly in contexts where other methods face important limitations. For example, retrospective medical record audits often suffer from recording bias and inadequate adjustment for case-mix variations [3]. Clinical vignettes, while useful for assessing HCP knowledge, may not accurately reflect actual practice [4]. Both vignettes and direct observations can be affected by the Hawthorne effect, where HCPs alter behavior when they know they are being observed [4]. Similarly, patient exit interviews may be influenced by social desirability and recall bias [5, 6]. In contrast, employing covert SPs allows researchers to observe HCP behavior in a natural environment with minimal Hawthorne effect or other biases [7]. This, along with strong internal validity and efficiency, makes the SP method a valuable tool for health services researchers [8].

Existing evidence syntheses have primarily focused on SP methodology in pharmacy practice research, emphasizing its value in assessing medication counseling, advice provision, and other pharmacist-led activities [7, 9–12]. While SP methodology is being increasingly adopted in pharmaceutical services research worldwide, particularly in community settings, recurring shortcomings persist. These include the infrequent provision of educational feedback and inconsistencies in reporting practices [10, 11].

Compared to pharmacy practice, SP methodology has been relatively underexplored in other healthcare contexts. The most comprehensive evidence synthesis in this area dates back nearly two decades and focused on studies where SPs assessed physicians’ performance in real practice settings [13]. Most of these studies were conducted in primary care settings, covering a broad range of clinical scenarios. Similar to findings in pharmaceutical research, the authors acknowledge that while SP methodology provides valuable insights into real-world practice, it has limitations that need to be addressed. They emphasize the need for clearer methodologies, more rigorous reporting, and greater awareness of potential selection bias. Additionally, they emphasized the importance of structured feedback mechanisms in improving HCP performance and maximizing the impact of SP assessments.

The limited attention given by reviewers to SP outside of pharmacy practice may be due to the broader scope of these studies, which span diverse healthcare settings, professions, and specialties. This heterogeneity could make it more challenging to compare findings and synthesize evidence than in pharmacy practice. However, the expansion of universal health coverage and the increasing focus on integrated care systems have reshaped healthcare delivery worldwide in recent decades [14–16]. As healthcare continues to evolve, SP methodology serves as a valuable tool for examining how these developments impact accessibility, quality, and other aspects of care across diverse healthcare environments. Despite potential challenges posed by study heterogeneity, synthesizing recent evidence can offer new methodological insights into SP research, identify emerging trends, and highlight areas requiring further development.

## Review questions

This scoping review aimed to explore the key characteristics of SP methodology applied in non-pharmaceutical health services research, answering the following review questions:

1. Where and in what settings have SP studies in non-pharmaceutical health services research been conducted (includes geographical distribution, healthcare settings, and provider specialties and professions)?
2. How are SPs utilized in studies (examines the number of SPs and SP-HCP contacts, the types of conditions used in scenarios, and types of interaction)?
3. Whether HCPs are aware of potential SP assessments and receive feedback?
4. How are ethical considerations and approval processes reported in SP studies?

## Methods

We conducted this scoping review in accordance with the JBI (formerly Joanna Briggs Institute) [17] methodological guidance for scoping reviews. The Preferred Reporting Items for Systematic Reviews and Meta-Analysis Protocols Extension for Scoping Reviews (PRISMA-ScR) were followed [18]. A protocol of this scoping review was registered on Open Science Framework (https://osf.io/8z9tn).

### Deviations from the protocol

There were several deviations from the a priori published protocol, all of which were made due to the high number of reports meeting our inclusion criteria. These amendments were necessary to align with the resources available for conducting this work.

First, we did not extract data on: (i) data collection period, (ii) gender, age, and work experience of HCPs, (iii) sample size (number of participated HCPs), (iv) age, gender, and ethnic background of SPs, (v) duration of the SP-HCP interaction, (vi) study design, (vii) information on how SPs were trained, (viii) study funding, and (ix) authors’ conclusions. However, we did extract data on ethical approval processes, recognizing this as an important aspect of SP methodology during the full-text screening.

Second, following independent piloting of data extraction on a sample of five reports by two reviewers, data extraction was completed by a single reviewer and then verified by another, rather than through the independent data extraction process stated in the protocol.

### Inclusion criteria

#### Participants

For this review, we considered reports involving HCPs interacting with SPs in non-pharmaceutical settings. We did not apply any criteria regarding the age, gender, profession, or specialty of the HCPs, nor the age or gender of the SPs. However, HCPs had to be fully trained professionals, not individuals still undergoing professional training.

#### Concept

We included reports of health services research studies on the covert use of the SPs, assessing HCPs’ behavior, skills and decision-making in real-world settings. We also considered reports where HCPs were informed that a study might take place but were not told exactly when. We did not apply any criteria regarding the type of HCP-SP interactions.

#### Context

We considered reports of studies that apply SP methodology in any geographical location and clinical setting (e.g., practices, clinics, hospitals), excluding reports limited to pharmacy settings. Reports involving multiple healthcare settings were eligible if at least one setting was non-pharmaceutical.

#### Types of sources

We focused on identifying reports of primary studies published in peer-reviewed journals, regardless of study design, within MEDLINE and Embase. Specifically, we considered for inclusion reports of studies with experimental, observational, qualitative, or mixed-methods designs. Other types of publications, such as reviews, editorials, and commentaries, were excluded. Furthermore, we did not consider grey literature or other non-peer-reviewed sources, such as magazine articles or blogs.

### Search strategy

A search strategy for this scoping review consisted of two blocks. The first block focused on the SP methodology, while the second block targeted the settings where the studies were conducted. To develop the first block for MEDLINE (PubMed), AP conducted a search terms frequency analysis using the quanteda R package, based on a set of 119 reports included in the systematic review on the use of SP methodology in pharmacy practice research [10]. The terms were analyzed within the title, abstract, and MeSH (medical subject headings) fields. Single words, as well as terms consisting of two and three words that appeared in more than two records, were considered potentially relevant for the first block of the search strategy. After examining the potentially relevant search terms, AP developed the first block of the search strategy.

The second block for MEDLINE (PubMed) was developed using exclusively MeSH terms referring to the settings relevant to our research question (e.g., hospitals [MeSH], inpatients [MeSH], outpatients [MeSH] etc.). We did not search within other fields, as expanding the search to other fields would have resulted in an overwhelming number of records, which screening was not feasible given the resource constraints. The resulting MEDLINE (PubMed) was assessed by the team, using the Peer Review of Electronic Search Strategies (PRESS) guideline [19], and then translated for Embase (Elsevier). To exclude records indexed in MEDLINE from the Embase (Elsevier) search results, and therefore to reduce the number of duplicates, we added the MEDLINE limit to the translated search strategy using the NOT Boolean operator (i.e., NOT [medline]/lim).

Furthermore, our search strategy focused on identifying reports published from May 2004 (as the search in the systematic review of Rethans et al. [13] was performed in April 2004). We searched MEDLINE (PubMed) and Embase (Elsevier) on October 11, 2023. Reports in English and German were considered for inclusion, a decision driven by our team’s language proficiency, as well as the lack of funding for professional translation or access to licensed software for reliable automated translation. We did not supplement the database search with other approaches (e.g., citation chasing) due to high number of included reports. The full search strategy for each database is presented in Additional file 1.

### Selection of reports

We uploaded all identified records into the EndNote reference management tool. Two reviewers (AP and NK) independently screened the titles and abstracts of the records against the inclusion criteria using the Covidence software [20]. Before this, AP and NK conducted a pilot test on a random sample of 5% of the total number of records. The results of the pilot test were discussed with the whole team. Afterwards, AP and NK continued screening the titles and abstracts of the remaining records. In the next step, AP and NK independently screened potentially eligible reports, documenting the reasons for excluding reports. Disagreements between the reviewers at any stage were resolved through discussion between the two reviewers, or by involving an additional reviewer (BP, DP or JB).

### Data extraction

A data-extraction form was developed by the research team, using Microsoft Excel. For piloting, AP and NK independently extracted the data on a sample of five reports. The results of the pilot test were discussed with the whole team to decide whether amendments were necessary. Data extraction was then completed by a single reviewer (AP or AMC) and verified by another reviewer (AP, JB, BP, NK, or AMC). Disagreements between the reviewers were resolved through discussion between the two reviewers, or by involving an additional reviewer (AP, JB, BP, NK, or AMC). No authors were contacted to request additional data.

We extracted data on the following elements:

- First Author
- Title
- Year
- Country/countries where the study was conducted (for the final results table we grouped the countries by continents)
- Number of SPs used in the study
- Number of SP-HCP contacts
- Settings (categorized using the wording applied in reports. However, we did not differentiate between types or specialties of clinics, health centers, hospitals or practices. For instance, urban, rural, private, and public hospitals were all categorized as 'hospitals')
- Specialty of HCPs (categorized according to the list of specialties recognized in the European Union and European Economic Area [21])
- Profession of HCPs (categorized using the wording applied in reports)
- ICD-10 chapter and code of the scenario applied (categorized according to the 10th revision of the International Statistical Classification of Diseases and Related Health Problems [22])
- Form of SP-HCP interaction
- Information on whether HCPs were informed about being assessed by the SPs without being notified of the specific timing
- Information on whether HCPs received feedback
- Information on whether the study was subject to ethics commission approval and the outcome of that decision. We categorized the data into three groups: (a) approved – reports that stated ethics committee approval was obtained, with or without a waiver for informed consent from HCPs; (b) not necessary – reports in which authors stated that ethics approval was not required, either based on the committee’s decision or in accordance with the country’s legal regulations; (c) not reported – reports that provided no information on ethical considerations

For settings, specialty, profession, ICD-10 chapter and code, and type of SP-HCP interaction, we reported the most commonly occurring categories, while any remaining groups with fewer than ten occurrences were categorized as ‘other’. To ensure consistency, the categorization criteria were piloted on a sample of five reports by AP and NK, and refinements were made based on team discussions before full data extraction.

The final version of the charting form is presented in Additional file 2.

### Data analysis and presentation

We presented the data in tables, detailing the characteristics of all included reports, and further stratified these characteristics by continent and HCP specialty. This choice was based on our assessment of the most meaningful distinctions while also considering the need to balance analytical depth with feasibility. We deemed continent and HCP specialty to be the most relevant stratification variables, as differences in healthcare infrastructure, regulatory environments, and cultural norms across continents can shape SP studies, influencing the mode of SP-HCP interactions, encounter settings, and ethical study requirements. Similarly, at the specialty level, stratification captures differences in how SP methodology is adapted to specific medical fields. Therefore, we assumed that these two variables capture much of the variation in SP studies while maintaining a manageable scope of analysis.

We expressed categorical variables as counts and percentages, and continuous variables as counts, medians, and interquartile ranges (IQR). Additionally, we visualized the trend in the number of publications over time with a figure. A narrative summary accompanies the results presented in the tables and figure.

## Results

### Study inclusion

**Fig. 1:**
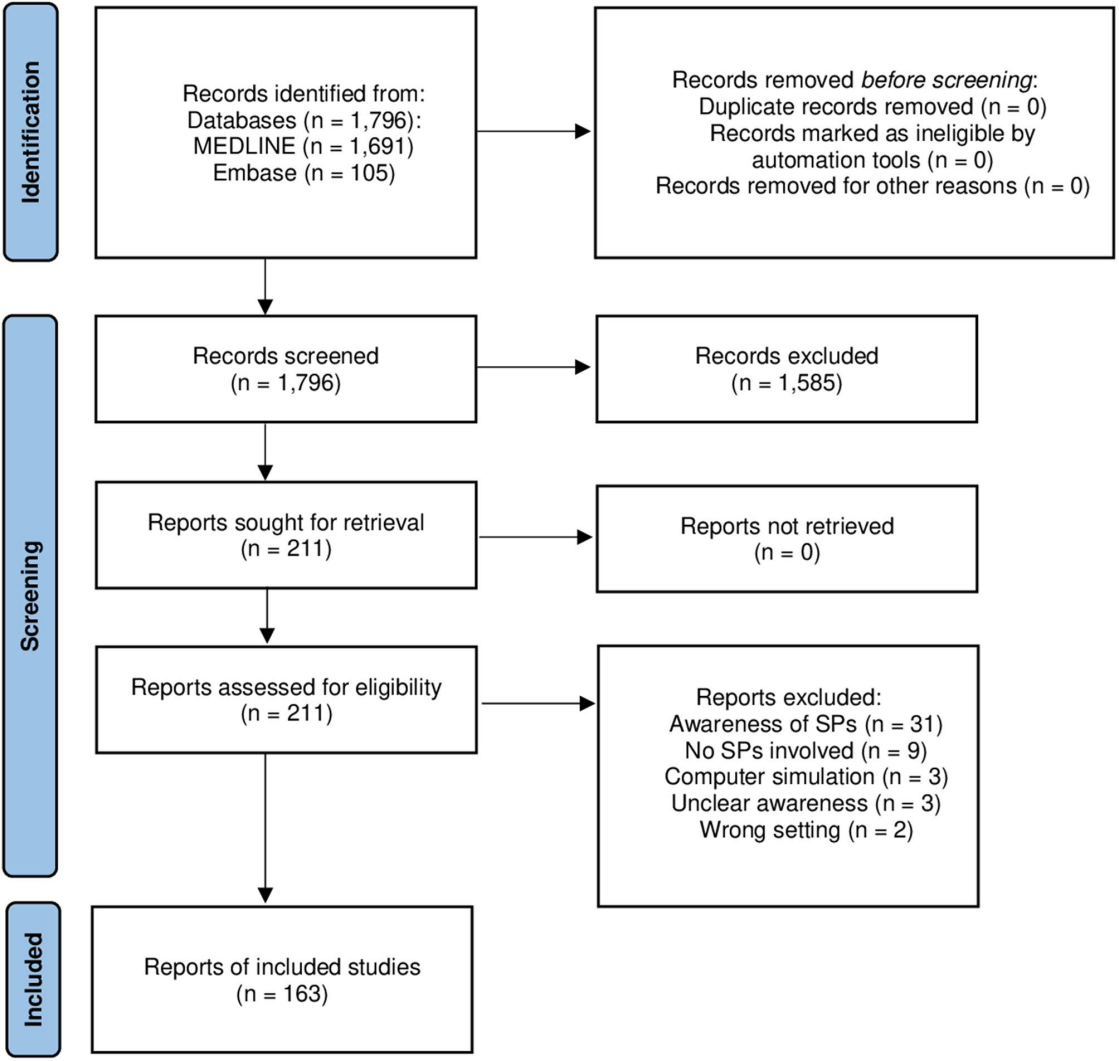
PRISMA 2020 flow diagram for new systematic reviews which included searches of databases and registers only

The search strategy for this scoping review yielded a total of 1,796 records (1,691 records sourced from PubMed and 105 from Embase). No duplicates were identified manually or by Covidence. After title and abstract screening, 1,582 records were excluded, leaving 214 reports for full-text screening. Ultimately, 163 reports in English were included after excluding 51 reports in full-text screening, with no eligible reports identified in German (Fig. 1).

### Review findings

**Fig. 2:**
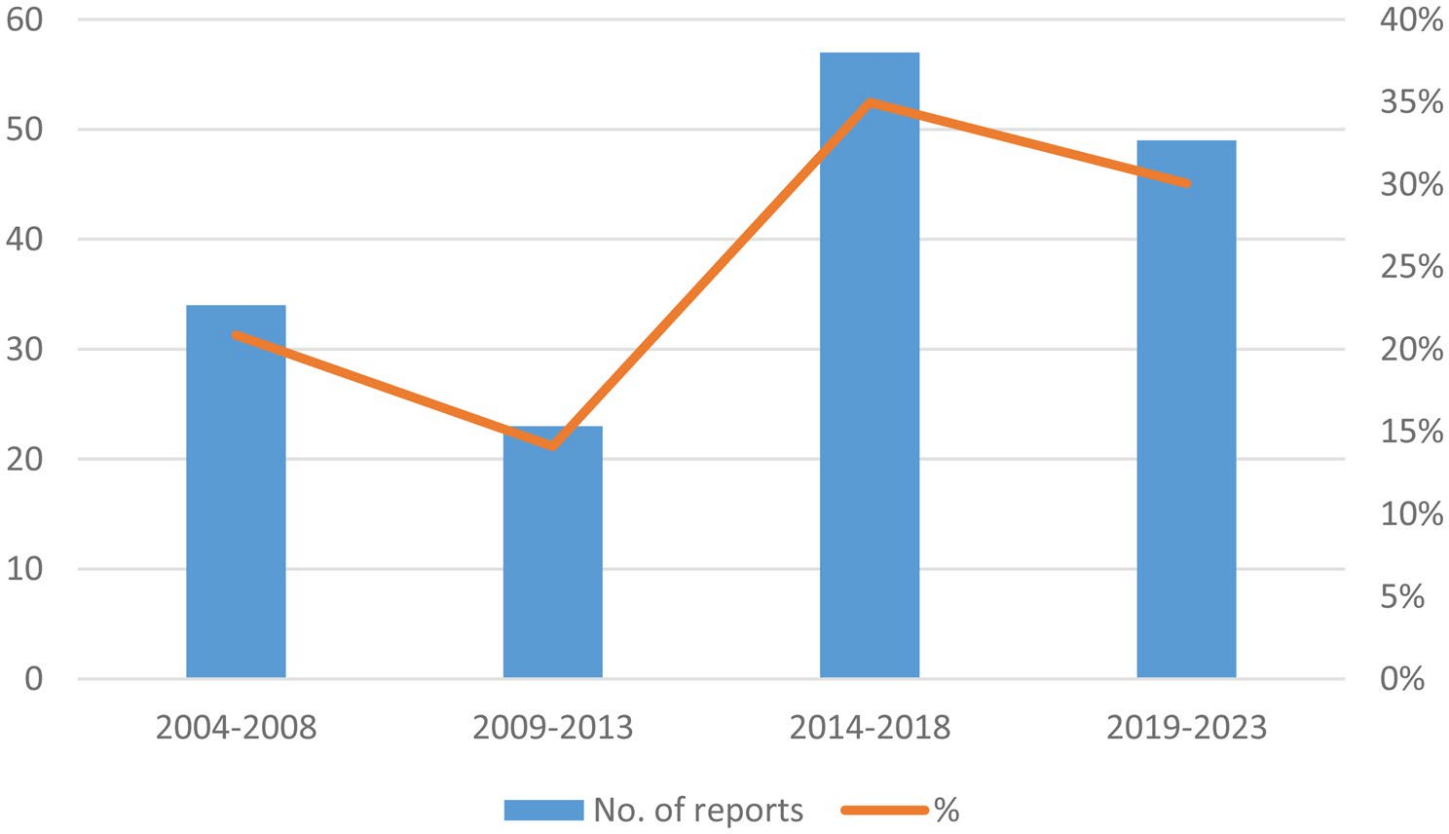
Reports published per five-year period

During the whole period from 2004 to 2023, there was a noticeable increase in the number of published reports, particularly between 2014 and 2018, which accounted for 35% of the total reports. This upward trend persisted from 2019 to 2023, despite a slight decrease, contributing 30% of the total reports (Fig. 2).

The majority of reports originated from North America (63.8%), with Africa and Asia each contributing 12.9%, and Europe 9.2% (Table 1). The median number of contacts between SPs and HCPs per report was 202.5, with an IQR of 99.3 to 528. The reports involved in total 1,249 SPs, with a median of 8 SPs per report (IQR: 3.8 to 15). The encounters primarily took place in clinics (39.9%) and practices (36.8%), with the rest occurring in hospitals (19.6%), health centers (12.9%), and other healthcare settings (19.0%), such as dispensaries, pharmacies, nursing homes each of which appeared in fewer than ten reports. The scenarios enacted by the SPs addressed various medical issues, categorized by ICD-10 chapters, with chapter *XXI (factors influencing health status and contact with health services)* being the most common (30.7%). The most frequently enacted scenarios related to the code *Z30 (contraceptive management)*, appearing in 13.5% of the reports.

Interactions between SPs and HCPs were mostly face-to-face (58.9%), with 42.9% occurring via telephone. A substantial proportion of the reports did not specify whether HCPs were informed about the study (42.9%) or received feedback post-interaction (90.8%). In 8.6% of the reports, it was stated that HCPs were not informed about the study, and in 1.8%, they did not receive feedback. Additionally, many reports lacked details on the specialties (41.1%) and professions (46.0%) of the HCPs involved, as well as on the ethics approval process (12.3%). When details on specialties were provided, family and general medicine (33.1%) and internal medicine (27.6%) were the most frequently reported. Other reported specialties included obstetrics and gynecology (8.6%) and oncology (6.1%). The remaining specialties, grouped under ‘Other’ (20.9%), encompassed a variety of fields such as pediatrics, accident and emergency medicine, orthopedics and other specialties that appeared less frequently. Regarding professions, physicians (41.7%), nurses (12.3%), and receptionists (7.4%) were the most commonly involved.

In terms of ethical considerations, 62.1% of reports indicated that the ethics committee approved the studies, while in 19.6% it was mentioned that such approval was not necessary. Additionally, 12.3% of reports did not provide any information on the ethics approval process.

**Table 1:** Characteristics of included reports

Characteristics*	
**No. of reports**	163
**Continent**	
Africa	21/163 (12.9%)
Asia	21/163 (12.9%)
Europe	15/163 (9.2%)
North America	104/163 (63.8%)
Other	4/163 (2.5%)
**No. of contacts**	
Total	153,328
Median	202.5
IQR (Q1-Q3)	428.8 (99.3–528)
**No. of SPs**	
Total	1249
Median	8
IQR (Q1-Q3)	11.3 (3.8–15)
**Settings**	
Clinics	65/163 (39.9%)
Health centers	21/163 (12.9%)
Hospitals	32/163 (19.6%)
Practices	60/163 (36.8%)
Other	31/163 (19.0%)
**Specialty**	
Not reported	67/163 (41.1%)
Family and general medicine	54/163 (33.1%)
Internal medicine	45/163 (27.6%)
Obstetrics and gynecology	14/163 (8.6%)
Oncology	10/163 (6.1%)
Other	34/163 (20.9%)
**Profession of HCP**	
Not reported	75/163 (46.0%)
Nurses	20/163 (12.3%)
Physicians	68/163 (41.7%)
Receptionists	12/163 (7.4%)
Other	23/163 (14.1%)
ICD-10 chapter*	
I	17/163 (10.4%)
V	18/163 (11.0%)
IX	13/163 (8.0%)
X	13/163 (8.0%)
XIII	13/163 (8.0%)
XVIII	29/163 (17.8%)
XXI	50/163 (30.7%)
Other	61/163 (37.4%)
ICD-10 code**	
Not reported	10/163 (6.1%)
F32 or F33	10/163 (6.1%)
R03	10/163 (6.1%)
Z00	10/163 (6.1%)
Z30	22/163 (13.5%)
Other	118/163 (72.4%)
**Type of interaction between SP and HCP**	
Face to face	96/163 (58.9%)
Telephone	70/163 (42.9%)
Other	4/163 (2.5%)
**HCPs were informed about the study**	
Not reported	70/163 (42.9%)
Yes	79/163 (48.5%)
No	14/163 (8.6%)
**HCPs received feedback after the study**	
Not reported	148/163 (90.8%)
Yes	12/163 (7.4%)
No	3/163 (1.8%)
**Ethics**	
Not reported	20/163 (12.3%)
Approved	111/163 (62.1%)
Not necessary	32/163 (19.6%)

* For several variables, multiple options were possible for each report, meaning studies could be conducted across various settings, involve multiple specialties or professions, and utilize different types of interactions

IQR = interquartile range; Q1 = quartile 1; Q3 = quartile 3, HCP = health care provider, SP = simulated patient

* ICD-10 chapters: I – certain infectious and parasitic diseases; V – mental and behavioural disorders; IX – diseases of the circulatory system; X – diseases of the respiratory system; XIII – diseases of the musculoskeletal system and connective tissue; XVIII – symptoms signs and abnormal clinical and laboratory findings not elsewhere classified; XXI – factors influencing health status and contact with health services

** ICD-10 codes: F32 or F33 – F32 depressive episode or F33 recurrent depressive disorder; R03 – abnormal blood pressure reading without diagnosis; Z00 – general examination and investigation of persons without complaint and reported-diagnosis; Z30 – contraceptive management

### Characteristics of SP encounters across continents

When stratifying results by the continent of report origin, several trends emerge. Clinics were the most common setting in Africa and Asia, each accounting for 52.4% of encounters, while practices were predominant in North America (48.1%) and Europe (46.7%) (Additional file 3). Specialty reporting shows that a high proportion of encounters in Africa (76.2%) and Asia (81.0%) did not specify the specialty HCPs, unlike in North America (27.9%) and Europe (40.0%) where internal medicine (42.3% in North America) and family and general medicine (46.7% in Europe) were more commonly reported. Profession-wise, SP encounters in Asia and Europe most frequently assessed physicians (61.9% and 33.3%, respectively), whereas the reporting of the HCP profession was often lacking in Africa (61.9%) and North America (50.0%).

When examining ICD-10 chapters, chapter *XXI (factors influencing health status and contact with health services)* was the most frequently reported in Africa (66.7%), whereas chapter *I (certain infectious and parasitic diseases)*, was the most common in Asia (52.4%). Additionally, the ICD-10 code *Z30 (contraceptive management)* was most commonly reported in Africa (52.4%). In North America, three codes were mostly reported: *F32/F33 (depressive episode/recurrent depressive disorder)*, *R03 (abnormal blood pressure reading without diagnosis)*, and *Z00 (general examination and investigation of persons without complaint and reported-diagnosis)*.

Face-to-face interactions were most common in Africa (100.0%) and Asia (90.5%), while telephone interactions dominated in North America (55.8%). Awareness that a study involving SP encounters might take place among HCPs was highest in Asia (61.9%), whereas most reports from Europe did not provide information on this (53.8%). Reports from all continents predominantly showed a lack of reporting on post-study feedback to HCPs, with the non-reporting rates highest in Asia (95.2%). Information on the ethics approval processes was mostly provided in reports from Africa (95.5%), with the lowest reporting in Asia (81%).

### Characteristics of SP encounters across medical specialties

Examining the nuances of SP encounters across various medical specialties reveals unique patterns and practices. For instance, the specialty of HCPs was mostly not reported for North America (43.3%) and Asia (25.4%) (Additional file 4). Additionally, 100% of reports on oncology and 97.8% on internal medicine were concentrated in North America. Regarding settings, clinics were most commonly used for internal medicine (44.4%) and obstetrics and gynecology (42.9%). Practices were the primary setting for family and general medicine (64.8%) and internal medicine (53.3%). Hospitals were notably used in oncology (40%). Physicians dominated across specialties, especially in family and general medicine (66.7%) and internal medicine (64.4%). Obstetrics and gynecology had a higher percentage of encounters involving nurses (28.6%).

Most encounters were face-to-face, except for obstetrics and gynecology and oncology, which had telephone interactions (71.4% and 50%, respectively). The findings indicate that in 68.5% of reports on family and general medicine, and 62.2% on internal medicine, HCPs were informed that SP study might be conducted. In contrast, 71.4% of reports on obstetrics and gynecology did not specify whether this information was provided to HCPs. Feedback to HCPs post-study was rarely reported across all specialties, with the highest rate of feedback being provided in oncology (10%). In terms of ethics approval processes, reporting was strongest in obstetrics and gynecology, with 92.8% of reports including this information, compared to 82.3% of reports in internal medicine.

## Discussion

This scoping review sought to examine the application of the SP methodology in non-pharmaceutical healthcare settings over the past two decades. Rather than evaluating the effectiveness of SP interventions, our primary objective was to map the use of SPs across various contexts, and therefore, we did not investigate specific outcomes. Our findings underline the diverse use of SPs across various geographic regions, clinical settings, and specialties, highlighting both the strengths and areas for improvement in this research methodology.

In the past twenty years, the number of reports has grown considerably, rising from 57 between 2004 and 2013 to 106 in the following decade. This trend suggests a growing interest in this method within health services research. Furthermore, in contrast to the earlier systematic review [12] that exclusively identified studies from North America and Europe, our scoping review reveals a growing application of SP methodology in Africa, Asia, and other regions, underscoring its broader global adoption over the last two decades.

However, despite this global expansion, the majority of reports came from North America (63.8%), which was somewhat unexpected, particularly given the SR findings on the use of SP methodology in pharmaceutical settings, where reports were more evenly distributed across continents [10]. One factor contributing to the higher number of studies in North America could be the implementation of the Patient Protection and Affordable Care Act [23]. Passed in 2010 by the US Congress, this policy aimed to expand Medicaid eligibility for low-income, non-elderly individuals [24]. In the years following its enactment, the SP methodology has been frequently utilized to evaluate Medicaid beneficiaries’ access to care, as reflected in several reports included in our scoping review [25–29] This, coupled with the longer history of SP methodology use in the USA, where the first study employing this approach was conducted over half a century ago, can further explain the extensive use and deep integration of SP methodology in North American healthcare research.

Our scoping review revealed that face-to-face interactions were the sole mode used in Africa (100%) and the primary approach in Asia (90.5%). In contrast, Europe and North America relied mostly on telephone contact with HCPs. The preference for face-to-face interactions in Africa and Asia may be driven by the necessity for direct consultations for specific scenarios chosen for the SPs encounters in these regions (e.g., contraceptive management). Furthermore, this preference may also be attributed to healthcare users’ favoring face-to-face interactions with HCPs [30], as well as the technological and infrastructural challenges present in some countries that hinder conducting telephone consultations [31].

Clinics and practices emerged as the primary settings for SP encounters, consistent with their roles as frontline healthcare facilities where patient interactions frequently occur. The relatively lower utilization of hospitals likely reflects the complexity and logistical challenges of integrating SPs into this environments, as well as the higher potential for SPs to be detected in such settings.

A notable limitation in the included reports was the frequent lack of detailed information on the specialties of HCPs (41.1%) and their professions (46.0%). This lack of specificity hinders comprehensive conclusions about the applicability of SP interactions across different medical fields and professional roles.

Another important finding was the lack of reporting on post-interaction feedback provided to HCPs, with only 7.4% of reports specifying that the feedback was provided. Compared to the findings of the systematic review of Rethans et al. [13], which indicated that feedback was provided in 17.5% of cases, our review found a considerably lower rate of feedback provision. Feedback is crucial for the educational aspect of SP methodology, as it helps HCPs recognize areas of their practice that do not meet expected standards and offers guidance on how to enhance their performance [32]. The fact that most reports do not mention feedback suggests that feedback may not have been provided, which represents a missed opportunity for enhancing HCP skills and patient care quality.

In approximately half of the reports, it was either explicitly stated that HCPs were not informed that an SP study might be conducted, or there was no information provided on whether they were informed. This raises ethical concerns, particularly regarding the process of obtaining informed consent from HCPs. One explanation for these findings is the argument that obtaining informed consent in SP studies could undermine the study’s scientific validity. The awareness of being covertly assessed might alter HCPs’ behavior, thus compromising the real-world setting of the study [33]. Furthermore, obtaining informed consent could introduce a selection bias, which becomes greater the more HCPs deny participation [12]. Therefore, some authors suggest that in cases where obtaining informed consent could compromise the authenticity of the data, waiving consent might be ethically justified to preserve the study’s validity and ensure that the research produces reliable and socially valuable results [33].

These challenges in balancing informed consent and scientific validity could contribute to the variability in ethical considerations observed across reports included in our scoping review. While most reports indicated that ethics approval was obtained, there was inconsistency regarding informed consent, as some approvals included a waiver for informed consent from HCPs, while others did not. Additionally, 32 reports stated that ethics approval was deemed unnecessary. Furthermore, 20 reports lacked information on ethical approval processes. Our findings align with those of a systematic review of SP methodology in pharmacy practice research, which also revealed such variability and lack of reporting on ethics approval [10].

The inconsistency in ethical considerations, coupled with the lack of comprehensive reporting highlights the need for clearer guidelines and standards in SP research. Although several manuals address the use of SPs in educational contexts [34–36], handbooks or guidelines specifically for applying SPs in healthcare services research seem to be lacking. However, an important step towards improving reporting practices was recently taken with the development and refinement of the Checklist for Reporting Research Using a Simulated Patient Methodology in Health (CRiSPHe) [37, 38]. CRiSPHe outlines a range of reporting criteria, including those related to ethical and organizational approvals, how healthcare providers were informed about or consented to covert assessment, and procedures following SP encounters, such as debriefing or feedback, which were among the areas most frequently underreported in our review [37, 38]. Continuing these efforts to develop manuals guiding SP research could greatly contribute to ensuring its coherence, transparency and ethical integrity.

Overall, our review aligns with previous research, emphasizing that the key challenges of SP methodology persist across diverse settings rather than being limited to a single context. Along with the authors of previous evidence syntheses, we stress the need for consistent reporting and standardized methodology in SP studies [7, 11]. The latter is particularly important, as it provides researchers with a framework for determining when and in what form HCPs can benefit from post-study feedback, as well as in which cases ethical approval is necessary. At the same time, our findings highlight the increasing use of SP research in assessing various aspects of healthcare across different specialties and professions, demonstrating that more researchers choose this methodology for its advantages over other research methods. Furthermore, our review extends prior evidence by illustrating that SP methodology can be effectively adapted to diverse healthcare environments in both high-income and low- and middle-income countries [9–11]. This adaptability highlights its potential to bridge research gaps in settings where traditional evaluation methods may be less feasible due to resource constraints. Additionally, as healthcare continue to evolve, the flexibility of SP methodology enables its use in emerging areas of research, such as telemedicine. However, to make the most of its potential, it is important to improve methodological consistency and follow existing reporting standards that promote transparency and allow for better comparison across studies.

### Limitations

This scoping review has several limitations that should be acknowledged. First, we excluded reports not available in English or German and limited our search to only two databases, MEDLINE (PubMed) and Embase, without performing citation chasing. These choices may have resulted in the omission of relevant reports, potentially affecting the comprehensiveness of our review.

Second, our unit of analysis was the report rather than the study due to the difficulty in linking reports to specific studies. The same issue was mentioned by Rethans et al. [13]. As noted in their work, it became clear that multiple reports referred to the same project only by cross-referencing the number of SPs, cases, or study participants, since many projects did not cite related reports. We did not adopt this approach due to the much higher number of identified reports in our review. By examining individual reports instead, we gained insights into reporting practices within studies using SP methodology.

Additionally, we did not extract information on study designs, as our primary focus was on characteristics directly reflecting the implementation of SP methodology, including SP-HCP interactions, settings, scenarios, and provider specialties. However, future reviews may benefit from examining study designs to explore how SP methodology is applied within different research approaches in health services research.

Finally, by restricting our review to reports in English and German and excluding additional sources such as institutional reports or other grey literature, we may have overlooked important perspectives on SP methodology. For example, language restrictions could partly explain the underrepresentation of reports from regions outside North America. A similar issue applies to source selection, as reports published on institutional websites or by professional organizations are likely to be in the local language. As a result, both restrictions may have limited our ability to capture a complete picture of regional differences in the application of SP methodology. Further research could benefit from including reports from a broader range of sources and languages to provide an even more comprehensive and globally representative understanding of SP methodology.

## Conclusions

This scoping review demonstrates the increasing and diverse application of the SP methodology across different regions, clinical settings, and specialties. We also identified several areas that require improvement, including the need for enhanced reporting practices and greater consistency in ethical considerations for SP studies. Developing methodological manuals and guidelines will address these gaps and improve the reliability of SP methodology, enabling studies that can lead to enhanced practices among HCPs and higher quality of care.

## Supplementary Information

Below is the link to the electronic supplementary material.

Supplementary Material 1

Supplementary Material 2

Supplementary Material 3

Supplementary Material 4

## Data Availability

The datasets used and/or analysed during the current study are available from the corresponding author on reasonable request.

## Abbreviations

CRiSPHe: Checklist for reporting research using a simulated patient methodology in Health
HCP: Health care provider
ICD-10: The 10th revision of the International Statistical Classification of Diseases and Related Health Problems
IQR: Interquartile range
JBI: Joanna Briggs Institute
PRISMA-ScR: Preferred Reporting Items for Systematic Reviews and Meta-Analysis Protocols Extension for Scoping Reviews
SP: Simulated patient
US/USA: United States of America

## References

[1] Siew AL, Wong JW, Chan EY. Effectiveness of simulated patients in geriatric education: A scoping review. Nurse Educ Today. 2021;100:104856. 33740706 10.1016/j.nedt.2021.104856

[2] Rosenhan DL. On being sane in insane places. Science. 1973;179(4070):250–8. 4683124 10.1126/science.179.4070.250

[3] Peabody JW, Luck J, Glassman P, Dresselhaus TR, Lee M. Comparison of vignettes, standardized patients, and chart abstraction: a prospective validation study of 3 methods for measuring quality. JAMA. 2000;283(13):1715–22. 10755498 10.1001/jama.283.13.1715

[4] Mohanan M, Vera-Hernández M, Das V, Giardili S, Goldhaber-Fiebert JD, Rabin TL, et al. The know-do gap in quality of health care for childhood diarrhea and pneumonia in rural India. JAMA Pediatr. 2015;169(4):349–57. 25686357 10.1001/jamapediatrics.2014.3445 PMC5023324

[5] Onishi J, Gupta S, Peters DH. Comparative analysis of exit interviews and direct clinical observations in pediatric ambulatory care services in Afghanistan. Int J Qual Health Care. 2011;23(1):76–82. 21131382 10.1093/intqhc/mzq074

[6] Sikombe K, Pry JM, Mody A, Rice B, Bukankala C, Eshun-Wilson I, et al. Comparison of patient exit interviews with unannounced standardised patients for assessing HIV service delivery in Zambia: a study nested within a cluster randomised trial. BMJ Open. 2023;13(7):e069086. 37407057 10.1136/bmjopen-2022-069086 PMC10335575

[7] Xu T, de Almeida Neto AC, Moles RJ. A systematic review of simulated-patient methods used in community pharmacy to assess the provision of non-prescription medicines. Int J Pharm Pract. 2012;20(5):307–19. 22953770 10.1111/j.2042-7174.2012.00201.x

[8] Collins JC, Chong WW, de Almeida Neto AC, Moles RJ, Schneider CR. The simulated patient method: Design and application in health services research. Res Social Adm Pharm. 2021;17(12):2108–15. 33972178 10.1016/j.sapharm.2021.04.021

[9] Watson MC, Norris P, Granas A. A systematic review of the use of simulated patients and pharmacy practice research. Int J Pharm Pract. 2006;14(2):83–93. 10.1111/ijpp.12570 31397533

[10] Björnsdottir I, Granas AG, Bradley A, Norris P. A systematic review of the use of simulated patient methodology in pharmacy practice research from 2006 to 2016. Int J Pharm Pract. 2020;28(1):13–25. 31397533 10.1111/ijpp.12570

[11] Boura F, Al-Tabakha M, Hassan N, Darwich M. Critical appraisal of simulated patient methodology to assess the practice of community pharmacist in the Middle East and North Africa region: A systematic review. Pharm Pract (Granada). 2022;20(3):2701. 36733522 10.18549/PharmPract.2022.3.2701 PMC9851829

[12] Kunow C, Langer B. Using the simulated patient methodology to assess the quality of counselling in german community pharmacies: a systematic review from 2005 TO 2018. Int J Pharm Pharm Sci. 2021;13(1):10–9.

[13] Rethans JJ, Gorter S, Bokken L, Morrison L. Unannounced standardised patients in real practice: a systematic literature review. Med Educ. 2007;41(6):537–49. 17518833 10.1111/j.1365-2929.2006.02689.x

[14] Kuramoto F. The Affordable Care Act and integrated care. J Soc Work Disabil Rehabil. 2014;13(1–2):44–86. 24329106 10.1080/1536710X.2013.870515

[15] Balicer R, Shadmi E, Manor O, Leventer-Roberts M, Israel. Structural and Functional Integration at the Israeli Healthcare System. In: Amelung V, Stein V, Suter E, Goodwin N, Nolte E, Balicer R, editors. Handbook Integrated Care. Cham: Springer International Publishing; 2021. pp. 1055–63.

[16] Castro MC, Massuda A, Almeida G, Menezes-Filho NA, Andrade MV, de Souza Noronha KVM, et al. Brazil’s unified health system: the first 30 years and prospects for the future. Lancet. 2019;394(10195):345–56. 31303318 10.1016/S0140-6736(19)31243-7

[17] Peters MDJ, Marnie C, Tricco AC, Pollock D, Munn Z, Alexander L, et al. Updated methodological guidance for the conduct of scoping reviews. JBI Evid Implement. 2021;19(1):3–10. 33570328 10.1097/XEB.0000000000000277

[18] Tricco AC, Lillie E, Zarin W, O’Brien KK, Colquhoun H, Levac D, et al. PRISMA Extension for Scoping Reviews (PRISMA-ScR): Checklist and Explanation. Ann Intern Med. 2018;169(7):467–73. 30178033 10.7326/M18-0850

[19] McGowan J, Sampson M, Salzwedel DM, Cogo E, Foerster V, Lefebvre C. PRESS Peer Review of Electronic Search Strategies: 2015 Guideline Statement. J Clin Epidemiol. 2016;75:40–6. 27005575 10.1016/j.jclinepi.2016.01.021

[20] Covidence systematic review software. Melbourne, Australia: Veritas Health innovation.

[21] Parliament TE, the Council of the European U. Directive 2005/36/EC of the European Parliament and of the Council of 7 September 2005 on the recognition of professional qualifications (Text with EEA relevance). Official Journal of the European Union. 2005.

[22] Organization WH. International Statistical classification of diseases and related health problems. 10th revision. 2016. Available from: https://icd.who.int/browse10/2016/en.

[23] Congress US. H. Patient Protection and Affordable Care Act, H.R. 3590, 111th Congress, 2nd session, enrolled bill. Washington: U.S. Government publishing office. 2010.

[24] Leszinsky L, Candon M. Primary Care Appointments for Medicaid Beneficiaries With Advanced Practitioners. Ann Fam Med. 2019;17(4):363–6. 31285214 10.1370/afm.2399 PMC6827647

[25] Benitez JA, Tipirneni R, Perez V, Davis MM. Does Primary Care Provider Supply Influence Medicaid Acceptability? Med Care. 2019;57(5):348–52. 30870393 10.1097/MLR.0000000000001110

[26] Chaudhry SB, Armbrecht ES, Shin Y, Matula S, Caffrey C, Varade R, et al. Pediatric access to dermatologists: Medicaid versus private insurance. J Am Acad Dermatol. 2013;68(5):738–48. 23474423 10.1016/j.jaad.2012.10.034

[27] Hsiang WR, Yousman L, Kim D, Cavallo JA, Kenney PA, Motamedinia P, et al. Access to Urologic Care at Urgent Care Centers. Urology. 2021;156:124–8. 34181971 10.1016/j.urology.2021.06.009

[28] Laditi F, Nie J, Hsiang W, Umer W, Haleem A, Marks V, et al. Access to urologic cancer care for Medicaid-insured patients. Urol Oncol. 2023;41(4):206.e21-.e27. 10.1016/j.urolonc.2023.01.014 36740488

[29] Marks VA, Hsiang WR, Nie J, Demkowicz P, Umer W, Haleem A, et al. Acceptance of Simulated Adult Patients With Medicaid Insurance Seeking Care in a Cancer Hospital for a New Cancer Diagnosis. JAMA Netw Open. 2022;5(7):e2222214. 35838668 10.1001/jamanetworkopen.2022.22214 PMC9287756

[30] Okoroafor IJ, Chukwuneke FN, Ifebunandu N, Onyeka TC, Ekwueme CO, Agwuna KK. Telemedicine and biomedical care in Africa: Prospects and challenges. Niger J Clin Pract. 2017;20(1):1–5. 27958238 10.4103/1119-3077.180065

[31] Fongwen NT, Nchafack A, Tetuh KM, Ong JJ, Tucker JD, Hughes G, et al. Telephone hotlines for infectious disease outbreaks in Africa: A review and qualitative study. J Public Health Afr. 2024;15(1):608. 39145288 10.4102/jphia.v15i1.608 PMC11321129

[32] Collins JC, Schneider CR, Naughtin CL, Wilson F, de Almeida Neto AC, Moles RJ. Mystery shopping and coaching as a form of audit and feedback to improve community pharmacy management of non-prescription medicine requests: an intervention study. BMJ Open. 2017;7(12):e019462. 29247115 10.1136/bmjopen-2017-019462 PMC5735410

[33] Rhodes KV, Miller FG. Simulated patient studies: an ethical analysis. Milbank Q. 2012;90(4):706–24. 23216428 10.1111/j.1468-0009.2012.00680.x PMC3530739

[34] Dudley F. The simulated patient handbook: a comprehensive guide for facilitators and simulated patients. CRC. 2018.

[35] Gliva-McConvey G, Nicholas CF, Clark L. Comprehensive healthcare simulation: implementing best practices in standardized patient methodology. Springer. 2020.

[36] Nestel D, Bearman M. Simulated patient methodology: theory, evidence and practice. Wiley. 2014.

[37] Amaratunge S, Harrison M, Clifford R, Seubert L, Page A, Bond C. Developing a checklist for reporting research using simulated patient methodology (CRiSP): a consensus study. Int J Pharm Pract. 2021;29(3):218–27. 33792718 10.1093/ijpp/riaa002

[38] Park JS, Page A, Clifford R, Bond C, Seubert L. Refining the CRiSPHe (checklist for reporting research using a simulated patient methodology in Health): a Delphi study. Int J Pharm Pract. 2024;32(4):322–8. 38752525 10.1093/ijpp/riae019

