# Supplementary material for: Simulated patient methodology applied in health services research: a scoping review
Source: BMC Health Serv Res. 2026 Mar 26;26:494. doi: 10.1186/s12913-026-14407-3 (PMC13063682; doi:10.1186/s12913-026-14407-3)
Supplement: Supplementary file 1 — Supplementary Material 1 [file 12913_2026_14407_MOESM1_ESM.docx]

**Simulated patient methodology applied in health services research: a scoping review**

**Electronic search terms and syntax**

**PubMed**

((("simulated patient"[Title/Abstract:~2] OR "simulated patients"[Title/Abstract:~2] OR "simulated client"[Title/Abstract:~2] OR "simulated clients"[Title/Abstract:~2] OR "simulated user"[Title/Abstract:~2] OR "simulated users"[Title/Abstract:~2] OR "mystery patient"[Title/Abstract:~2] OR "mystery patients"[Title/Abstract:~2] OR "mystery client"[Title/Abstract:~2] OR "mystery clients"[Title/Abstract:~2] OR "mystery shopper"[Title/Abstract:~2] OR "mystery shoppers"[Title/Abstract:~2] OR "secret shopper"[Title/Abstract:~2] OR "secret shoppers"[Title/Abstract:~2] OR "pseudo patient"[Title/Abstract:~2] OR "pseudo patients"[Title/Abstract:~2] OR "standardized patient"[Title/Abstract:~2] OR "standardized patients"[Title/Abstract:~2] OR "standardised patient"[Title/Abstract:~2] OR "standardised patients"[Title/Abstract:~2] OR "pseudo patient"[Title/Abstract:~2] OR "pseudo patients"[Title/Abstract:~2] OR "patient simulation"[MeSH Terms]) AND ("hospitals"[MeSH Terms] OR "inpatients"[MeSH Terms] OR "outpatients"[MeSH Terms] OR "outpatient clinics, hospital"[MeSH Terms] OR "ambulatory care"[MeSH Terms] OR "ambulatory care facilities"[MeSH Terms] OR "ambulatory surgical procedures"[MeSH Terms] OR "primary health care"[MeSH Terms] OR "office visits"[MeSH Terms] OR ("primary nursing"[MeSH Terms] OR "primary care nursing"[MeSH Terms]) OR "physicians, primary care"[MeSH Terms] OR "private practice"[MeSH Terms] OR "group practice"[MeSH Terms] OR "partnership practice"[MeSH Terms] OR "community health services"[MeSH Terms])) NOT ("case reports"[Publication Type] OR "letter"[Publication Type] OR "editorial"[Publication Type] OR "comment"[Publication Type] OR "news"[Publication Type] OR "preprint"[Publication Type] OR "historical article"[Publication Type] OR "interview"[Publication Type])) AND 2004/05/01:3000/12/31[Date - Publication]

Date of search: 11.10.2023
No. of hits: 1691

**Embase**

('simulated near/2 patient*':ti,ab OR 'simulated near/2 client*':ti,ab OR 'simulated near/2 user*':ti,ab OR 'mystery near/2 patient*':ti,ab OR 'mystery near/2 client*':ti,ab OR 'mystery near/2 shopper*':ti,ab OR 'secret near/2 shopper*':ti,ab OR 'standardized near/2 patient*':ti,ab OR 'standardised near/2 patient*':ti,ab OR 'pseudo near/2 patient*':ti,ab OR 'patient simulation'/exp) AND ('hospital'/exp OR 'hospital patient'/exp OR 'outpatient'/exp OR 'outpatient department'/exp OR 'ambulatory care'/exp OR 'ambulatory surgery'/exp OR 'primary health care'/exp OR 'primary nursing'/exp OR 'general practitioner'/exp OR 'private practice'/exp OR 'group practice'/exp OR 'professional practice'/exp OR 'community care'/exp) AND [embase]/lim NOT [medline]/lim NOT ('case reports':pt OR 'letter':pt OR 'editorial':pt OR 'comment':pt OR 'news':pt OR 'preprint':pt OR 'historical article':pt OR 'interview':pt) AND [01-05-2004]/sd

Date of search: 11.10.2023
No. of hits: 105
