# Supplementary material for: Simulated patient methodology applied in health services research: a scoping review
Source: BMC Health Serv Res. 2026 Mar 26;26:494. doi: 10.1186/s12913-026-14407-3 (PMC13063682; doi:10.1186/s12913-026-14407-3)
Supplement: Supplementary file 2 — Supplementary Material 2 [file 12913_2026_14407_MOESM2_ESM.docx]

Table 2. Characteristics of SP encounters within included reports by medical specialty of HCPs

| **Characteristics*** | **Family and general medicine (n=54)** | **Internal medicine (n=45)** | **Obstetrics and gynecology (n=14)** | **Oncology (n=10)** | **Other (n=34)** | **Not reported (n=67)** |
| --- | --- | --- | --- | --- | --- | --- |
| **No. of contacts** |  |  |  |  |  |  |
| Total | 65269 | 63340 | 3834 | 2965 | 15823 | 78402 |
| Median | 217 | 298 | 154 | 181 | 219 | 217.5 |
| IQR (Q1-Q3) | 403.5 (155-558.5) | 421 (178-599) | 491.5 (73.5-565) | 174 (160-334) | 521 (78-599) | 534.5 (120.7-655.3) |
| **No. of SPs** |  |  |  |  |  |  |
| Total | 357 | 350 | 59 | 63 | 208 | 741 |
| Median | 6 | 8 | 4 | 6 | 7.5 | 12 |
| IQR (Q1-Q3) | 10.75 (3-13.75) | 13.25 (4.8-18) | 6 (1-7) | 9.5 (4.5-14) | 11.3 (3.3-14.5) | 11 (6-17) |
| **Continent** |  |  |  |  |  |  |
| Africa | 2/54 (3.7%) | 0/45 (0%) | 2/14 (14.3%) | 0/10 (0%) | 1/34 (2.9%) | 16/67 (23.9%) |
| Asia | 1/54 (1.9%) | 1/45 (2.2%) | 3/14 (21.4%) | 0/10 (0%) | 2/34 (5.9%) | 17/67 (25.4%) |
| Europe | 7/54 (13.0%) | 0/45 (0%) | 0/14 (0%) | 0/10 (0%) | 2/34 (5.9%) | 6/67 (9.0%) |
| North America | 43/54 (79.6%) | 44/45 (97.8%) | 8/14 (57.1%) | 10/10 (100.0%) | 28/34 (82.4%) | 29/67 (43.3%) |
| Other | 1/54 (1.9%) | 0/45 (0%) | 1/14 (7.1%) | 0/10 (0%) | 1/34 (2.9%) | 1/67 (1.5%) |
| **Settings** |  |  |  |  |  |  |
| Clinics | 16/54 (29.6%) | 20/45 (44.4%) | 6/14 (42.9%) | 1/10 (10.0%) | 12/34 (35.3%) | 29/67 (43.3%) |
| Health centers | 1/54 (1.9%) | 0/45 (0%) | 2/14 (14.3%) | 0/10 (0%) | 0/34 (0%) | 19/67 (28.4%) |
| Hospitals | 1/54 (1.9%) | 0/45 (0%) | 3/14 (21.4%) | 4/10 (40.0%) | 7/34 (20.6%) | 19/67 (28.4%) |
| Practices | 35/54 (64.8%) | 24/45 (53.3%) | 4/14 (28.6%) | 5/10 (50.0%) | 13/34 (38.2%) | 17/67 (25.4%) |
| Other | 5/54 (9.3%) | 3/45 (6.7%) | 2/14 (14.3%) | 1/10 (10.0% | 6/34 (17.6%) | 22/67 (32.8%) |
| **Profession of HCP** |  |  |  |  |  |  |
| Not reported | 14/54 (25.9%) | 14/45 (31.1%) | 9/14 (64.3%) | 3/10 (30.0%) | 20/34 (58.8%) | 36/67 (53.7%) |
| Nurses | 5/54 (9.3%) | 2/45 (4.4%) | 4/14 (28.6%) | 0/10 (0%) | 5/34 (14.7%) | 10/67 (14.9%) |
| Physicians | 36/54 (66.7%) | 29/45 (64.4%) | 1/14 (7.1%) | 5/10 (50.0%) | 6/34 (17.6%) | 26/67 (38.8%) |
| Receptionists | 3/54 (5.6%) | 2/45 (4.4%) | 2/14 (14.3%) | 2/10 (20.0%) | 3/34 (8.8%) | 5/67 (7.5%) |
| Other | 3/54 (5.6%) | 4/45 (8.9%) | 4/14 (28.6%) | 1/10 (10.0%) | 9/34 (26.5%) | 10/67 (14.9%) |
| **ICD-10 chapter**** |  |  |  |  |  |  |
| I | 1/54 (1.9%) | 1/45 (2.2%) | 1/14 (7.1%) | 0/10 (0%) | 1/34 (2.9%) | 15/67 (22.4%) |
| V | 10/54 (18.5%) | 10/54 (22.2%) | 0/14 (0%) | 0/10 (0%) | 4/34 (11.8%) | 2/67 (3.0%) |
| IX | 1/54 (1.9%) | 0/45 (0%) | 0/14 (0%) | 0/10 (0%) | 0/34 (0%) | 12/67 (17.9%) |
| X | 4/54 (7.4%) | 3/45 (6.7%) | 0/14 (0%) | 0/10 (0%) | 4/34 (11.8%) | 7/67 (10.4%) |
| XIII | 8/54 (14.8%) | 9/45 (20.0%) | 1/14 (7.1%) | 0/10 (0%) | 3/34 (8.8%) | 1/67 (1.5%) |
| XVIII | 18/54 (33.3%) | 18/45 (40%) | 2/14 (14.3%) | 0/10 (0%) | 2/34 (5.9%) | 9/67 (13.4%) |
| XXI | 13/54 24.1%) | 10/45 (22.2%) | 8/14 (57.1%) | 0/10 (0%) | 9/34 (26.5%) | 25/67 (37.3%) |
| Other | 24/54 (44.4%) | 18/45 (40.0%) | 6/14 (42.9%) | 10/10 (100.0%) | 20/34 (58.8%) | 17/67 (25.4%) |
| **ICD-10 code***** |  |  |  |  |  |  |
| Not reported | 3/54 (5.6%) | 3/45 (6.7%) | 1/14 (7.1%) | 1/10 (10.0%) | 3/34 (8.8%) | 5/67 (7.5%) |
| F32 or F33 | 7/54 (13.0%) | 7/45 (15.6%) | 0/14 (0%) | 0/10 (0%) | 2/34 (5.9%) | 0/34 (0%) |
| R03 | 6/54 (11.1%) | 7/45 (15.6%) | 0/14 (0%) | 0/10 (0%) | 0/34 (0%) | 3/67 (4.5%) |
| Z00 | 7/54 (13.0%) | 7/45 (15.6%) | 0/14 (0%) | 0/10 (0%) | 3/34 (8.8%) | 3/67 (4.5%) |
| Z30 | 2/54 (3.7%) | 0/45 (0%) | 3/14 (21.4%) | 0/10 (0%) | 2/34 (5.9%) | 17/67 (25.4%) |
| Other | 42/54 77.8%) | 34/45 (75.6%) | 10/14 (71.4%) | 10/10 (100.0%) | 26/34 (76.5%) | 42/67 (62.7%) |
| **Type of interaction between SP and HCP** |  |  |  |  |  |  |
| Face to face | 35/54 (64.8%) | 29/45 (64.4%) | 5/14 (35.7%) | 5/10 (50.0%) | 9/34 (26.5%) | 47/67 (70.1%) |
| Telephone | 18/54 (33.3%) | 16/45 (35.6%) | 10/14 (71.4%) | 5/10 (50.0%) | 25/34 (73.5%) | 22/67 (32.8%) |
| Other | 3/54 (5.6%) | 2/45 (4.4%) | 0/14 (0%) | 0/10 (0%) | 3/34 (8.8%) | 1/67 (1.5%) |
| **HCPs were informed about the study** |  |  |  |  |  |  |
| Not reported | 14/54 (25.9%) | 15/45 (33.3%) | 10/14 (71,4%) | 5/10 (50.0%) | 20/34 (58.8%) | 31/67 (46.3%) |
| Yes | 37/54 (68.5%) | 28/45 (62.2%) | 2/14 (14.3%) | 5/10 (50.0%) | 7/34 (20.6%) | 30/67 (44.8%) |
| No | 3/54 (5.6%) | 2/45 (4.4%) | 2/14 (14.3%) | 0/10 (0%) | 7/34 (20.6%) | 6/67 (9.0%) |
| **HCPs received feedback after the study** |  |  |  |  |  |  |
| Not reported | 50/54 (92.6%) | 41/45 (91.1%) | 13/14 (92.9%) | 9/10 (90.0%) | 31/34 (91.2%) | 60/67 (89.6%) |
| Yes | 3/54 (7.4%) | 4/45 (8.9%) | 1/14 (7.1%) | 1/10 (10%) | 3/34 (8.8%) | 4/67 (6.0%) |
| No | 0/54 (0%) | 0/45 (0%) | 0/14 (0%) | 0/10 (0%) | 0/34 (0%) | 3/67 (4.5%) |
| **Ethics** |  |  |  |  |  |  |
| Not reported | 7/54 (13.0%) | 8/45 (17.8%) | 1/14 (7.1%) | 1/10 (10.0%) | 2/34 (5.9%) | 10/67 (14.9%) |
| Approved | 40/54 (74.1%) | 34/45 (75.6%) | 10/14 (71.4%) | 4/10 (40.0%) | 22/34 (64.7%) | 47/67 (70.1%) |
| Not necessary | 7/54 (13.0%) | 3/45 (6.7%) | 3/14 (21.4%) | 5/10 (50.0%) | 10/34 (29.4%) | 10/67 (14.9%) |

* For several variables, multiple options were possible for each report, meaning studies could be conducted across various settings, involve multiple specialties or professions, and utilize different types of interactions.
IQR = interquartile range; Q1 = quartile 1; Q3 = quartile 3
HCP = health care provider
SP = simulated patient
** ICD-10 chapters: I – certain infectious and parasitic diseases; V – mental and behavioural disorders; IX – diseases of the circulatory system; X – diseases of the respiratory system; XIII – diseases of the musculoskeletal system and connective tissue; XVIII – symptoms signs and abnormal clinical and laboratory findings not elsewhere classified; XXI – factors influencing health status and contact with health services.
*** ICD-10 codes: F32 or F33 – F32 depressive episode or F33 recurrent depressive disorder; R03 – abnormal blood pressure reading without diagnosis; Z00 – general examination and investigation of persons without complaint and reported-diagnosis; Z30 – contraceptive management.
