# Supplementary material for: Simulated patient methodology applied in health services research: a scoping review
Source: BMC Health Serv Res. 2026 Mar 26;26:494. doi: 10.1186/s12913-026-14407-3 (PMC13063682; doi:10.1186/s12913-026-14407-3)
Supplement: Supplementary file 3 — Supplementary Material 3 [file 12913_2026_14407_MOESM3_ESM.docx]

Table 3. Characteristics of SP encounters within included reports by continent

| **Characteristics*** | **Africa (n=21)** | **Asia (n=21)** | **Europe (n=15)** | **North America (n=104)** | **Other (n=4)** |
| --- | --- | --- | --- | --- | --- |
| **No. of contacts** |  |  |  |  |  |
| Total | 4477 | 7591 | 3765 | 136710 | 785 |
| Median | 180 | 428 | 100 | 218 | 47 |
| IQR (Q1-Q3) | 97.5 (128-225.5) | 358 (140-498) | 327 (30-357) | 473.5 (126.8-600.3) | 333 (41.5-374.5) |
| **No. of SPs** |  |  |  |  |  |
| Total | 182 | 366 | 136 | 558 | 19 |
| Median | 12 | 17 | 4 | 6 | 4 |
| IQR (Q1-Q3) | 7 (8-15) | 12.8 (8.5-21.3) | 12.8 (2.5-15.3) | 10.8 (3-13.8) | 5.2 (1.8-7) |
| **Settings** |  |  |  |  |  |
| Clinics | 11/21 (52.4%) | 11/21 (52.4%) | 5/15 (33.3%) | 39/104 (37.5%) | 1/4 (25.0%) |
| Health centers | 8/21 (38.1%) | 10/21 (47.6%) | 0/15 (0%) | 2/104 (1.9%) | 1/4 (25.0%) |
| Hospitals | 10/21 (47.6%) | 9/21 (42.9%) | 1/21 (6.7%) | 12/104 (11.5%) | 0/4 (0%) |
| Practices | 1/21 (4.8%) | 0/21 (0%) | 7/15 (46.7%) | 50/104 (48.1%) | 2/4 (50.0%) |
| Other | 14/21 (66.7%) | 2/21 (9.5%) | 4/15 (26.7%) | 11/104 (10.6%) | 0/4 (0%) |
| **Specialty of HCP** |  |  |  |  |  |
| Not reported | 16/21 (76.2%) | 17/21 (81.0%) | 6/15 (40.0%) | 29/104 (27.9%) | 1/4 (25.0%) |
| Family and general medicine | 2/21 (9.5%) | 1/21 (4.8%) | 7/15 (46.7%) | 43/104 (41.3%) | 1/4 (25.0%) |
| Internal medicine | 0/21 (0%) | 1/21 (4.8%) | 0/15 (0%) | 44/104 (42.3%) | 0/4 (0%) |
| Obstetrics and gynecology | 2/21 (9.5%) | 3/21 (14.3%) | 0/15 (0%) | 8/104 (7.7%) | 1/4 (25.0%) |
| Oncology | 0/21 (0%) | 0/21 (0%) | 0/15 (0%) | 10/104 (9.6%) | 0/4 (0%) |
| Other | 1/21 (4.8%) | 2/21 (9.5%) | 2/21 (13.3%) | 28/104 (26.9%) | 0/4 (0%) |
| **Profession of HCP** |  |  |  |  |  |
| Not reported | 13/21 (61.9%) | 7/21 (33.3%) | 3/15 (20.0%) | 52/104 (50.0%) | 2/4 (50.0%) |
| Nurses | 8/21 (38.1%) | 2/21 (9.5%) | 2/15 (13.3%) | 8/104 (7.7%) | 0/4 (0%) |
| Physicians | 4/21 (19%) | 13/21 (61.9%) | 5/15 (33.3%) | 45/104 (43.3%) | 1/4 (25.0%) |
| Receptionists | 2/21 (9.5%) | 2/21 (9.5%) | 4/15 (26.7%) | 4/104 (3.8%) | 0/4 (0%) |
| Other | 3/21 (14.3%) | 5/21 (23.8%) | 4/15 (26.7%) | 10/104 (9.6%) | 1/4(25.0%) |
| **ICD-10 chapter**** |  |  |  |  |  |
| I | 3/21 (14.3%) | 11/21 (52.4%) | 1/15 (6.7%) | 2/104 (1.9%) | 0/4 (0%) |
| V | 0/21 (0%) | 0/21 (0%) | 0/15 (0%) | 17/104 (16.3%) | 1/4 (25.0%) |
| IX | 1/21 (14.3%) | 9/21 (42.9%) | 1/15 (6.7%) | 2/104 (1.9%) | 0/4 (0%) |
| X | 2/21 (9.5%) | 7/21 (33.3%) | 0/15 (0%) | 3/104 (2.9%) | 1/4 (25.0%) |
| XIII | 0/21 (0%) | 0/21 (0%) | 2/15 (13.3%) | 11/104 (10.6%) | 0/4 (0%) |
| XVIII | 1/21 (4.8%) | 1/21 (4.8%) | 2/15 (13.3%) | 25/104 (24.0%) | 0/4 (0%) |
| XXI | 14/21 (66.7%) | 2/21 (9.5%) | 4/15 (26.7%) | 30/104 (28.8%) | 2/4 (50.0%) |
| Other | 2/21 (9.5%) | 5/21 (23.8%) | 5/15 (33.3%) | 48/104 (46.2%) | 1/4 (25.0%) |
| **ICD-10 code***** |  |  |  |  |  |
| Not reported | 0/21 (0%) | 1/21 (4.8%) | 3/15 (20.0%) | 6/104 (5.8%) | 0/4 (0%) |
| F32 or F33 | 0/21 (0%) | 0/21 (0%) | 0/15 (0%) | 10/104 (9.6%) | 0/4 (0%) |
| R03 | 0/21 (0%) | 0/21 (0%) | 0/15 (0%) | 10/104 (9.6%) | 0/4 (0%) |
| Z00 | 0/21 (0%) | 0/21 (0%) | 0/15 (0%) | 10/104 (9.6%) | 0/4 (0%) |
| Z30 | 11/21 (52.4%) | 2/21 (9.5%) | 3/15 (20%) | 6/104 (5.8%) | 2/4 (50.0%) |
| Other | 19/11 (47.6%) | 18/21 (85.7%) | 9/15 (60%) | 79/104 (76.0%) | 2/4 (50.0%) |
| **Type of interaction between SP and HCP** |  |  |  |  |  |
| Face to face | 21/21 (100.0%) | 19/21 (90.5%) | 9/15 (60%) | 46/104 (44.2%) | 3/4 (75.0%) |
| Telephone | 0/21 (0%) | 2/21 (9.5%) | 9/15 (60%) | 58/104 (55.8%) | 1/4 (25.0%) |
| Other | 0/21 (0%) | 0/21 (0%) | 1/15 (6.7%) | 3/104 (2.9%) | 0/4 (0%) |
| **HCPs were informed about the study** |  |  |  |  |  |
| Not reported | 5/21 (23.8%) | 6/21 (28.6%) | 3/15 (20.0%) | 56/104 (53.8%) | 0/4 (0%) |
| Yes | 13/21 (61.9%) | 13/21 (61.9%) | 12/15 (80.0%) | 40/104 (38.5%) | 3/4 (75.0%) |
| No | 3/21 (14.3%) | 1/21 (9.5%) | 0/15 (0%) | 8/104 (7.7.%) | 1/4 (25.0%) |
| **HCPs received feedback after the study** |  |  |  |  |  |
| Not reported | 18/21 (85.7%) | 20/21 (95.2%) | 13/15 (86.7%) | 95/104 (91.3%) | 4/4 (100.0%) |
| Yes | 1/21 (9.5%) | 1/21 (4.8%) | 2/15 (13.3%) | 8/104 (7.7%) | 0/4 (0%) |
| No | 2/21 (4.8%) | 0/21 (0%) | 0/15 (0%) | 1/104 (1.0%) | 0/4 (0%) |
| **Ethics** |  |  |  |  |  |
| Not reported | 1/21 (4.8%) | 4/21 (19.0%) | 2/15 (13.3%) | 13/104 (12.5%) | 0/4 (0%) |
| Approved | 18/21 (85.7%) | 17/21 (81.0%) | 10/15 (66.7%) | 64/104 (61.5%) | 4/4 (100.0%) |
| Not necessary | 2/21 (9.5%) | 0/21 (0%) | 3/15 (20.0%) | 27/104 (26.0%) | 0/4 (0%) |

* For several variables, multiple options were possible for each report, meaning studies could be conducted across various settings, involve multiple specialties or professions, and utilize different types of interactions.
IQR = interquartile range; Q1 = quartile 1; Q3 = quartile 3
HCP = health care provider
SP = simulated patient
** ICD-10 chapters: I – certain infectious and parasitic diseases; V – mental and behavioural disorders; IX – diseases of the circulatory system; X – diseases of the respiratory system; XIII – diseases of the musculoskeletal system and connective tissue; XVIII – symptoms signs and abnormal clinical and laboratory findings not elsewhere classified; XXI – factors influencing health status and contact with health services.
*** ICD-10 codes: F32 or F33 – F32 depressive episode or F33 recurrent depressive disorder; R03 – abnormal blood pressure reading without diagnosis; Z00 – general examination and investigation of persons without complaint and reported-diagnosis; Z30 – contraceptive management.
